# Comparative evaluation of leukocyte- and platelet-rich plasma and pure platelet-rich plasma for cartilage regeneration

**DOI:** 10.1038/srep43301

**Published:** 2017-03-07

**Authors:** Zhengliang Xu, Wenjing Yin, Yuelei Zhang, Xin Qi, Yixuan Chen, Xuetao Xie, Changqing Zhang

**Affiliations:** 1 Department of Orthopaedic Surgery, Shanghai Jiao Tong University Affiliated Sixth People’s Hospital, Shanghai, China

## Abstract

Platelet-rich plasma (PRP) has gained growing popularity in the treatment of articular cartilage lesions in the last decade. However, the potential harmful effects of leukocytes in PRP on cartilage regeneration have seldom been studied *in vitro*, and not at all *in vivo* yet. The objective of the present study is to compare the effects of leukocyte- and platelet-rich plasma (L-PRP) and pure platelet-rich plasma (P-PRP) on cartilage repair and NF-κB pathway, in order to explore the mechanism underlying the function of leukocytes in PRP in cartilage regeneration. The constituent analysis showed that P-PRP had significantly lower concentrations of leukocytes and pro-inflammatory cytokines compared with L-PRP. In addition, cell proliferation and differentiation assays indicated P-PRP promoted growth and chondrogenesis of rabbit bone marrow mesenchymal stem cells (rBMSC) significantly compared with L-PRP. Despite similarity in macroscopic appearance, the implantation of P-PRP combining rBMSC *in vivo* yielded better cartilage repair results than the L-PRP group based on histological examination. Importantly, the therapeutic effects of PRP on cartilage regeneration could be enhanced by removing leukocytes to avoid the activation of the NF-κB pathway. Thus, PRP without concentrated leukocytes may be more suitable for the treatment of articular cartilage lesions.

The repair capacity of articular cartilage is limited and large cartilage defects generally fail to heal spontaneously, making intervention necessary in order to avoid the progression of osteoarthritis^1^. Nevertheless, the therapeutic effect for large cartilage damage is not satisfactory, as autografts suffer from the inadequate tissue availability and the associated morbidity of the donor site, and allografts are limited by transplant rejection^2^,^3^. The tissue engineering for the treatment of articular cartilage defects presents a promising strategy^4^, however, many problems remain. For instance, natural materials suffer from the limitation of immunogenicity, potential risks of transmitting animal-originated pathogens, and weak mechanical properties^5^; synthetic materials lack natural sites for cell adhesion and may cause a local reduction in pH and potential inflammation due to the degradation through hydrolysis^6^. Moreover, scaffolds combining natural and synthetic materials barely show good results in *in vivo* studies^7^. Much attention has been paid to the biological safety and efficacy of the scaffolds^8^. It is safe and reliable when the scaffolds are obtained from the patient’s own tissue.

Platelet-rich plasma (PRP) is an autologous blood product containing concentrated platelets. After activation by thrombin or calcium chloride, the platelets in PRP release platelet-derived growth factor (PDGF), transforming growth factor (TGF), insulin-like growth factor (IGF), epidermal growth factor (EGF), vascular endothelial growth factor (VEGF), and many other growth factors through degranulation^9^,^10^. During cartilage formation or chondrocyte differentiation, TGF-β induces chondrogenesis of bone marrow-derived stem cells (BMSC), while PDGF aids chondrocytes to maintain the hyaline-like chondral phenotype and promote proliferation and proteoglycan synthesis^11^. In a previous study, we demonstrated that PRP gel presents a porous bioactive scaffold for cartilage repair^12^.

Despite the increasing use of PRP for cartilage lesions^13^,^14^, the optimal PRP formulation is still unknown, and over the past few years, much attention has been focused on the leukocyte concentrations in PRP. In a clinical study by Filardo^15^, leukocyte-rich PRP (L-PRP) resulted in a higher incidence of side effects in the treatment of osteoarthritis compared with pure PRP (P-PRP), which had a lower leukocyte concentration, possibly due to the fact that leukocytes in PRP may deliver pro-inflammatory cytokines, such as interleukin-1β (IL-1β) and tumor necrosis factor-α (TNF-α), at the site of injection, resulting in the production of destructive proteases that inhibit the formation and promote the degradation of extracellular matrix^15^,^16^,^17^. IL-1β and TNF-α induce deleterious effects through the nuclear factor κB (NF-κB) signalling pathway^18^,^19^. NF-κB proteins are typically present in an inactive form in the cytoplasm bound to IκB (an inhibitory protein). Cell stimulation through IL-1β or TNF-α leads to the nuclear translocation of NF-κB to trigger the expression of a wide range of regulatory genes involved in apoptosis, inflammation, and other immune responses^20^. Therefore, the concentrated leukocytes in L-PRP may activate the NF-κB pathway through IL-1β and TNF-α to inhibit tissue healing. However, this has not yet been substantiated.

BMSC are defined as self-renewal, multi-potent progenitor cells that differentiate into several mesenchymal lineages^21^, and they could be obtained in a less invasive procedure from autologous bone marrow compared with chondrocytes harvested from cartilage extraction. As a primary cell candidate for cartilage tissue engineering, BMSC has been extensively studied to repair osteochondral defects and enhance cartilage regeneration^22^,^23^.

The objective of the present study was to compare the effects of L-PRP and P-PRP (PRPs) on rabbit BMSC (rBMSC) *in vitro* and on cartilage repair *in vivo* and preliminarily explore the mechanism to improve the efficacy of PRP therapy.

## Results

### Components of PRPs and whole blood

**Figure 1:**
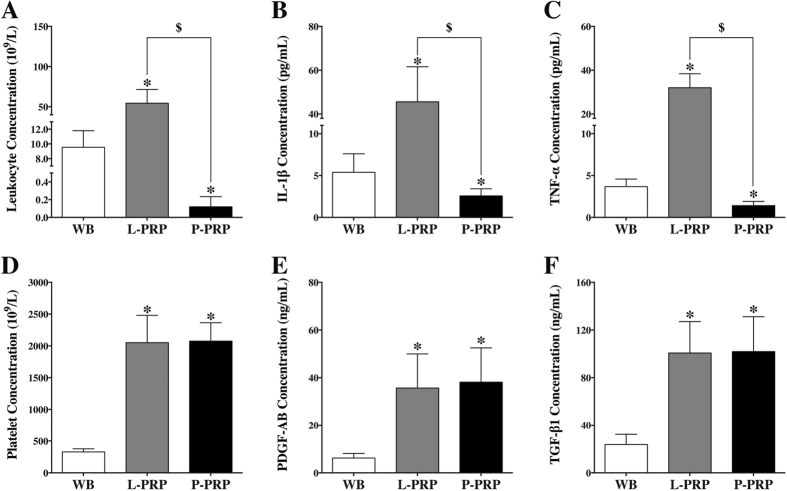
Cellular and cytokine concentrations in WB, L-PRP and P-PRP. Concentrations of leukocytes (**A**), IL-1β (**B**), and TNF-α (**C**) in P-PRP were significantly lower than those in L-PRP and WB; concentrations of platelets (**D**), PDGF-AB (**E**), and TGF-β1 (**F**) in L-PRP and P-PRP were similar, but significantly higher than those in WB. *p < 0.01 compared with WB; ^$^p < 0.01 L-PRP versus P-PRP; n = 10.

The components of PRPs and whole blood (WB) are shown in Fig. 1. The mean leukocyte concentration of L-PRP was significantly higher than that of whole blood, while P-PRP had a lower concentration than whole blood (p < 0.01, Fig. 1A). The levels of IL-1β and TNF-α were also higher in L-PRP and lower in P-PRP compared with whole blood (p < 0.01, Fig. 1B and C). L-PRP and P-PRP had a similar level of platelet concentration, and produced an almost 6-fold increase in platelet concentration over whole blood (p < 0.01, Fig. 1D). Moreover, the concentrations of PDGF-AB and TGF-β1 were similar between L-PRP and P-PRP (p > 0.01, Fig. 1E and F), but significantly lower in whole blood (p < 0.01).

**Figure 2:**
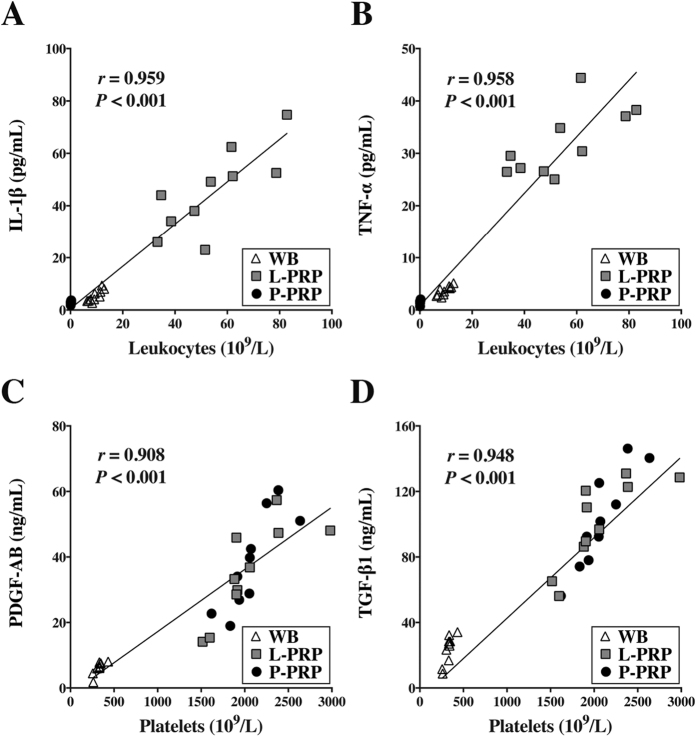
Correlations between cytokine concentrations and cellular components in WB, L-PRP and P-PRP. Positive correlations were observed between the concentrations of leukocytes and those of IL-1β (**A**) and TNF-α (**B**), and between the concentrations of platelets and those of PDGF-AB (**C**) and TGF-β1 (**D**) in WB, L-PRP and P-PRP; n = 30.

The correlation analysis showed that the leukocyte concentration was positively correlated with the concentrations of IL-1β (r = 0.959, p < 0.001, Fig. 2A) and TNF-α concentration (r = 0.958, p < 0.001, Fig. 2B), and the platelet concentration was significantly positively correlated with the concentrations of PDGF-AB (r = 0.908, p < 0.001, Fig. 2C) and TGF-β1 in the whole blood, L-PRP and P-PRP (r = 0.948, p < 0.001, Fig. 2D).

### Effects of PRPs on rBMSC

**Figure 3:**
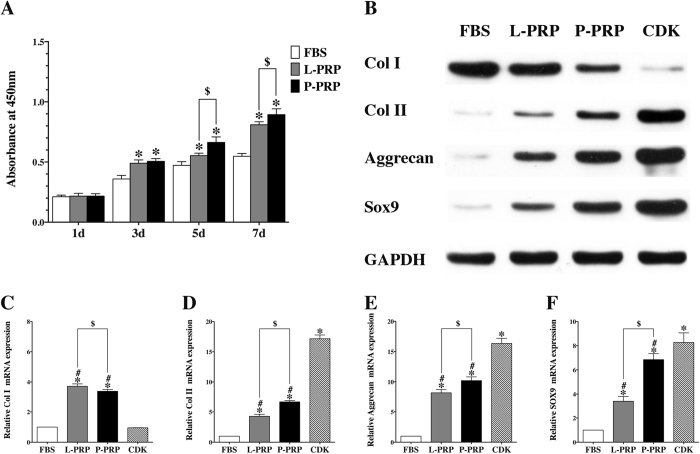
PRPs promoted proliferation and chondrogenetic differentiation of rBMSC. (**A**) CCK-8 assay showed that both L-PRP and P-PRP promoted rBMSC proliferation on day 3, 5 and 7 compared with FBS, but P-PRP showed greater effects on rBMSC proliferation on day 5 and 7 compared with L-PRP; (**B**) western blotting analysis revealed that P-PRP upregulated protein expression of SOX-9, Aggrecan, and Col II, and downregulated protein expression of Col I, compared with L-PRP and FBS; (**C–F**), qRT-PCR analysis demonstrated that both L-PRP and P-PRP upregulated mRNA expression of SOX-9, Aggrecan, Col II, and Col I compared with FBS, but P-PRP showed greater effects on mRNA expression of chondrogenic-related marker genes (SOX-9, Aggrecan, and Col II) and weaker effects on mRNA expression of osteogenic-related marker gene (Col I) compared with L-PRP. *p < 0.01 compared with WB; ^#^p < 0.01 compared with CDK; ^$^p < 0.01 L-PRP versus P-PRP; n = 3.

The trilineage differentiation and surface marker identification of rBMSC are shown in Figure S1(See the supplementary file). The Cell Counting Kit-8 (CCK-8) assay demonstrated that all groups presented good proliferation with no significant difference on the first day of culture (p > 0.01), while PRPs had better effects on rBMSC proliferation after 3, 5 and 7 days of culture than the fetal bovine serum (FBS) group. But the P-PRP group showed better effects on rBMSC proliferation than the L-PRP group after culture for 5 and 7 days (p < 0.01, Fig. 3A).

Western blot analysis showed that PRP groups had increased protein levels of Col II, Aggrecan and Sox9 compared with the FBS group, although the level of up-regulation was not as high as that in the CDK group. The results also showed that the expression levels were higher in the P-PRP group than those in the L-PRP group. The protein level of Col I was higher in the FBS group compared with other groups, and the L-PRP group showed higher Col I protein expression than the P-PRP group, while the CDK group showed the lowest expression (Fig. 3B).

The chondrogenic-related marker genes, including collagen type II (Col II), Aggrecan and Sox9, mRNA expression was significantly increased expression in the PRP groups than that in the FBS group. In addition, mRNA expression was significantly higher in the P-PRP group than in the L-PRP group, with the highest expression observed in the commercially available chondrogenesis differentiation kit (CDK) group (p < 0.01, Fig. 3D–F). The mRNA expression of collagen type I (Col I) was inhibited in the CDK group but increased in the PRP groups (p < 0.01, Fig. 3C).

### Effects of PRPs on NF-κB pathway

**Figure 4:**
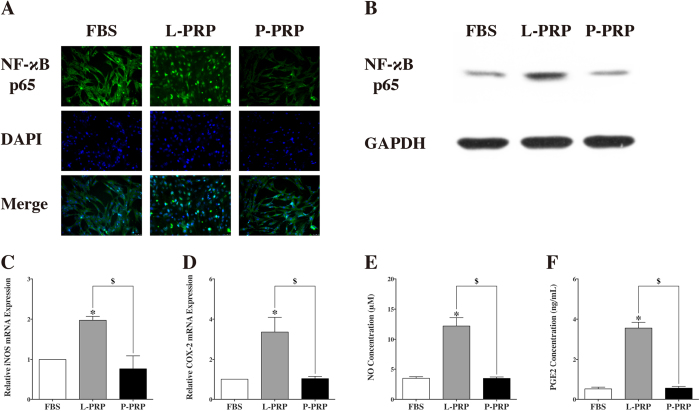
L-PRP induced the activation of NF-κB pathway. (**A**) Immunofluorescence staining revealed that NF-κB p65 was observed in the nucleus in the L-PRP group, and in the cytoplasm in the P-PRP and FBS groups; (**B**) western blotting analysis showed that L-PRP upregulated protein expression of NF-κB p65 in the nucleus compared with P-PRP and FBS; (**C** and **D**) qRT-PCR analysis showed that the mRNA expression levels of iNOS and COX-2 were significantly higher in the L-PRP group than those in the P-PRP and FBS groups; (**E** and **F**) Production of PGE2 and NO was significantly enhanced in the L-PRP group in comparison with that in the P-PRP and FBS groups. *p < 0.01 compared with WB; ^$^p < 0.01 L-PRP versus P-PRP; n = 3.

After culture for 1 hour, the immunofluorescence staining showed that NF-κB p65 was primarily located in the cytoplasm of cells treated with culture medium containing 10% FBS or P-PRP, but NF-κB p65 was located in the nuclei of cells treated with culture medium containing 10% L-PRP (Fig. 4A). Western blot analysis revealed that nuclear NF-κB p65 protein expression increased in the L-PRP group (Fig. 4B).

The qRT-PCR analysis showed that the L-PRP group showed higher inducible nitric oxide synthase (iNOS) and Cyclooxygenase-2 (COX-2) mRNA expression than the P-PRP and FBS groups (p < 0.01, Fig. 4C and D). The results of the nitric oxide (NO) production test demonstrated that the L-PRP group produced more NO than the P-PRP and FBS groups, and enzyme-linked immunosorbent assay (ELISA) indicated that the level of Prostaglandin E2 (PGE2) was significantly higher in the L-PRP group than in the P-PRP and FBS groups (p < 0.01, Fig. 4E and F). Moreover, the results indicated no significant difference between the P-PRP group and the FBS group (Fig. 4C–F).

### Macroscopic observations of cartilage repair

**Figure 5:**
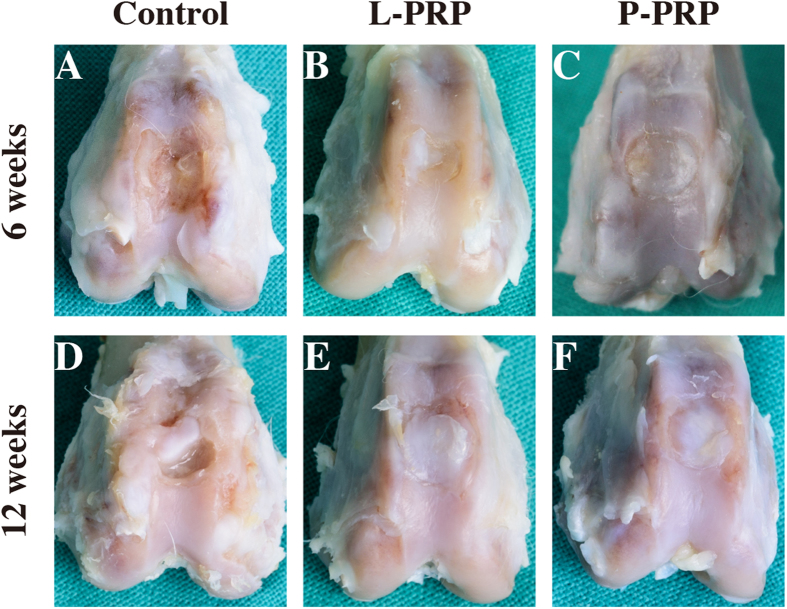
Effects of PRPs on cartilage repair in rabbits — gross observation. Macroscopic appearance of the cartilage healing in the control, L-PRP and P-PRP groups at 6 weeks (**A–C**), and 12 weeks (**D–F**) after implantation. n = 6.

At 6 weeks after surgery, macroscopic observation of the full-thickness cartilage defect sites appeared glossy white, and mostly well-integrated regenerated tissue in the P-PRP group, but the surface was not as smooth as the normal articular surface. Defects in the L-PRP group were filled with regenerated bone tissue halfway covered with whitish cartilage-like tissue. Defects in the control group were concave, filled with fibrous-like tissue, while the joints were slightly degenerated (Fig. 5A–C).

At 12 weeks postoperatively, the regenerated tissue seemed similar in the P-PRP group and the L-PRP group. Specifically, the regenerated tissue looks more opaque and closer to the adjacent cartilage tissue in the P-PRP group than that in the L-PRP group. In the control group, the defects were partially concave, filled with fibrous tissue; meanwhile the joints were moderately degenerated (Fig. 5D–F).

### Micro-computed tomography (Micro-CT) scanning analysis

**Figure 6:**
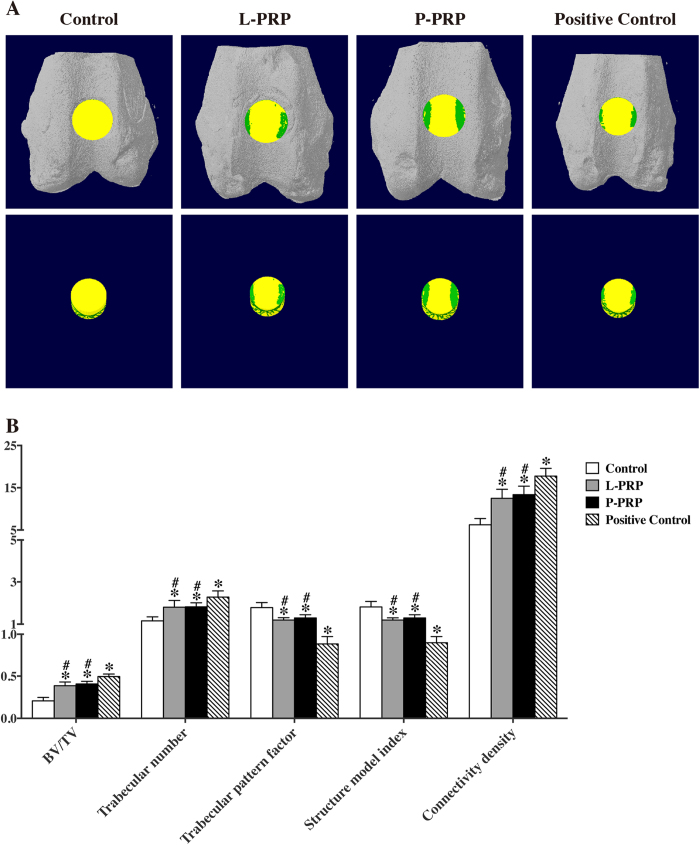
Effects of PRPs on osteochondral repair in rabbits — quantitative analysis of regenerated bone tissue using a 3-D Micro-CT model. (**A**) Region of interest (ROI) corresponding to the original defects in rabbits. The green colour represents regenerated bone, and the yellow colour represents other tissues in the original defect (top: 3-D models *in situ*; bottom: 3-D models *ex situ*). (**B**) Comparison of the bone regeneration in the 4 groups using the ratio of BV/TV, trabecular number, trabecular pattern factor, structure model index and connectivity density at 12 weeks. Non-treated rabbits were used as a positive control. BV, bone volume; TV, tissue volume; *p < 0.05 compared with the control group; ^#^p < 0.05 compared with the positive control group; n = 6.

Mineralized bone formation was assessed using Micro-CT scanning. Non-treated rabbits were used as a positive control, and representative pictures are shown in Fig. 6A. The Micro-CT parameter analysis showed that the values of BV/TV (bone volume/tissue volume) ratio, trabecular number, and connectivity density were significantly higher in the PRP and positive control groups compared with the control group at week 12 (p < 0.05, Fig. 6B). The values of trabecular pattern factor and structure model index in the control group were significantly higher than those in the other three groups. However, these values in the positive control group were significantly lower than those in the PRP groups (p < 0.05, Fig. 6B). However, the value of all 5 parameters was not significantly different between the L-PRP and P-PRP groups (p < 0.05, Fig. 6B).

### Histological findings

**Figure 7:**
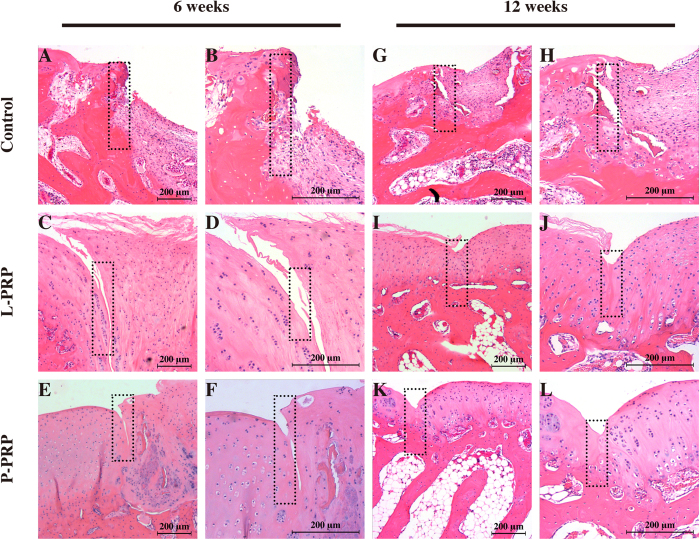
Effects of PRPs on cartilage repair in rabbits — H&E staining. Representative H&E stained sections in the control, L-PRP and P-PRP groups at 6 weeks (**A–F**) and 12 weeks (**G–L**). The rectangular squares demonstrated the conjunction between the regenerated tissue (right half) and the normal tissue (left half). n = 6.

The H&E staining showed that, at 6 weeks after surgery, the defects in the control group were primarily filled with fibrous tissue and remained concave (Fig. 7A and B); in the L-PRP group, the articular surface was partially filled with fibrous tissue, with moderate integration and splitting (Fig. 7C and D); in the P-PRP group, the joint surface was integrated relatively well with the regenerated tissue in the defects, with subchondral bone formation at the bottom and regenerated round cells on the top (Fig. 7E and F). At 12 weeks postoperativel, the joint surface was still concave in the defects covered with fibrous tissue in the control group, and little new bone formation was observed (Fig. 7G and H); in the L-PRP group, more cartilage-like tissue formation was observed, but the new regenerated tissue was thinner compared with the surrounding normal cartilage (Fig. 7I and J); the defects in the P-PRP group were fully filled with cartilage-like tissue, and well integrated with the normal cartilage, although the amount of regenerated cells was not as abundant as observed in normal cartilage (Fig. 7K and L).

**Figure 8:**
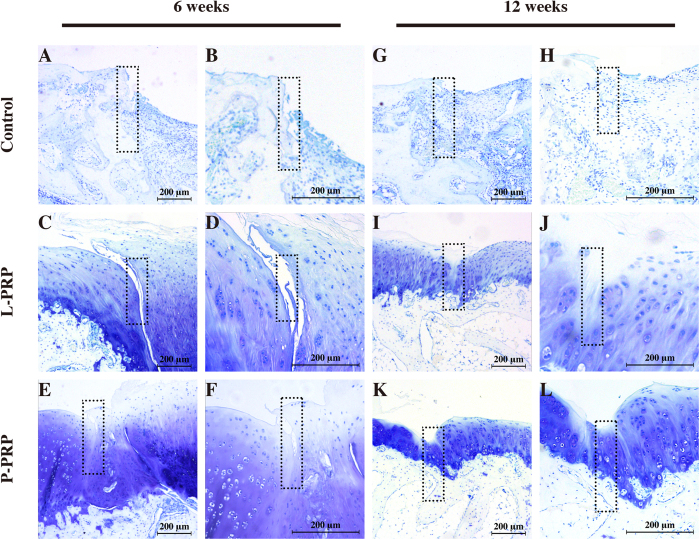
Effects of PRPs on cartilage repair in rabbits —toluidine blue staining. Representative toluidine blue stained sections in the control, L-PRP and P-PRP groups at 6 weeks (**A–F**) and 12 weeks (**G–L**). The rectangular squares demonstrated the conjunction between the regenerated tissue (right half) and the normal tissue (left half). n = 6.

The toluidine blue staining showed that, at 6 weeks after surgery, there was few cartilage matrix in the control group and much more in the PRP groups (Fig. 8A–F). However, a wide split was observed in the L-PRP group and a relatively narrow one was observed in the P-PRP group. Moreover, more cartilage matrix was observed in the P-PRP group compared with the L-PRP group (Fig. 8C–F). At 12 weeks, new bone formation was clearly better than that at 6 weeks in the control group, but few cartilage matrix was observed (Fig. 8G and H); a narrow split was still observed in the L-PRP group, but it was almost not seen in the P-PRP group (Fig. 8I–L); in addition, there was much more cartilage matrix in the P-PRP group compared with the L-PRP group.

**Figure 9:**
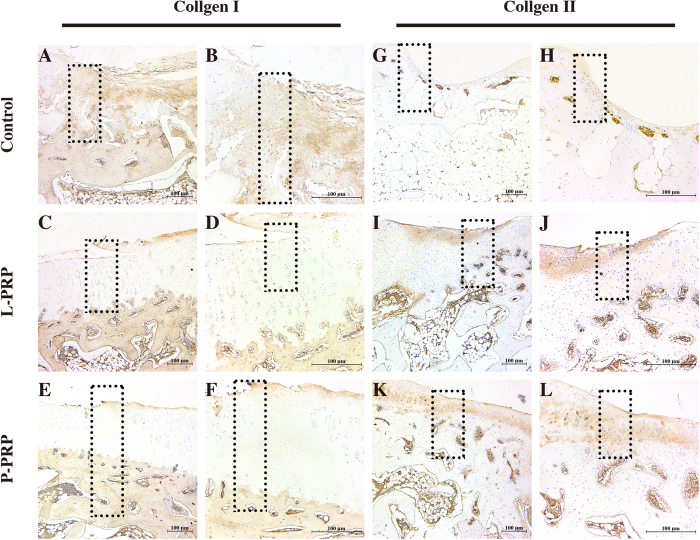
Effects of PRPs on cartilage repair in rabbits — immunohistochemical staining. Representative sections using Col I (**A–F**) and Col II (**G–L**) staining in the control, L-PRP and P-PRP groups at 12 weeks. The rectangular squares demonstrated the conjunction between the regenerated tissue (right half) and the normal tissue (left half). n = 6.

Immunohistochemical staining at 12 weeks showed that the content of Col I decreased in the regenerated tissue, while the content of Col II simultaneously increased in the following sequence: the control group, the L-PRP group and the P-PRP group (Fig. 9).

**Figure 10:**
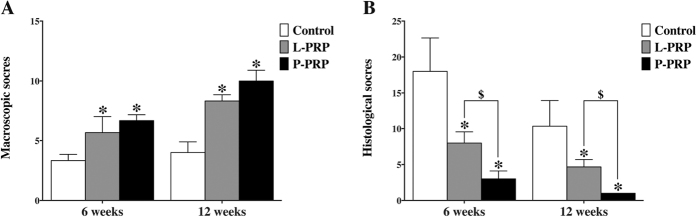
Effects of PRPs on cartilage repair in rabbits — macroscopic and histological scoring. (**A**) macroscopic ICRS scores; (**B**) histological OARSI scores. *p < 0.05 compared with the control group; ^$^p < 0.05 L-PRP versus P-PRP; n = 6.

International Cartilage Repair Society (ICRS) scoring for macroscopic evaluation of cartilage repair showed that the scores in the PRP groups were higher than that in the control group at week 6 and week 12. There was no significant difference between the L-PRP and P-PRP groups at week 6 or at week 12 (p > 0.05, Fig. 10A).

Osteoarthritis Research Society International (OARSI) scoring system was used to quantitatively analyze the histological evaluation. The results showed that the scores in the PRP groups were lower than that in the control group, and the scores in the P-PRP were lower than that in the L-PRP group at week 6 and 12 (p < 0.05, Fig. 10B). A low score means favorable outcome according to the OARSI scoring system.

## Discussion

The present study evaluated and compared the effects of L-PRP and P-PRP on cartilage regeneration and the NF-κB pathway. The findings showed that both L-PRP and P-PRP had ~6-fold platelet concentration and increased PDGF-AB and TGF-β1 concentrations compared with the whole blood. P-PRP, which had a significantly lower concentrations of leukocytes, IL-1β and TNF-α than L-PRP, presented better effects on rBMSC proliferation and chondrogenesis than L-PRP. In addition, L-PRP induced the nuclear translocation of NF-κB p65, increased the mRNA expression of iNOS and COX-2, and enhanced the production of NO and PGE2 in rBMSC, indicating that L-PRP strongly activated the NF-κB pathway. Moreover, rBMSC-containing P-PRP-derived scaffolds presented better cartilage healing results than rBMSC-containing L-PRP-derived scaffolds *in vivo*.

PRP treatment has become widespread in sports medicine due to the use of patient’s own blood product, ease of preparation, and beneficial effects on tissue repair^24^,^25^,^26^,^27^,^28^. However, the optimal method for preparing PRP for the treatment in cartilage repair is unknown, and different methods may result in different platelet and leukocyte concentrations, potentially affecting cell responses^29^. PDGF induces articular cartilage anabolism, chondrocyte proliferation and stem cell migration to the joint’s injured tissues^30^, and TGF-β1 increases the cartilage extracellular matrix (ECM) synthesis, diminishes joint inflammation and promotes the differentiation of synovial membrane stem cells into chondrocytes^31^. In the present study, PRPs released more growth factors, approximately 6-fold higher than the baseline level. From the third day, rBMSC cultured with PRPs presented better proliferation than the FBS group, reflecting the increased concentration of growth factors. Shapiro *et al*. reported that chondrocytes from the native cartilage did not participate in the repopulation of the defect, and that the cartilaginous repair appeared to be mediated by the proliferation and differentiation of mesenchymal cells from the bone marrow^32^. TNF-α was selected as a pro-inflammatory cytokine, and this protein along with IL-1β are key cytokines associated with the catabolic state observed in OA^33^, and leukocytes are the primary source of inflammatory cytokines such as IL-1 and TNF-α^34^,^35^,^36^,^37^,^38^. L-PRP contained more leukocytes, TNF-α and IL-1β than P-PRP in the present study. The cell proliferation assay showed that the L-PRP group had less cells than the P-PRP group after 5 and 7 days of culture, likely reflecting the harmful effects of TNF-α and IL-1β. This finding is consistent with the study of Anitua *et al*.^35^, showing that fibroblasts respond differently to various PRP formulations.

Recent *in vitro* evidence suggests that P-PRP treatment could be more suitable for tendon^17^,^39^ and joint^40^,^41^,^42^ injuries than L-PRP, as the lower leukocyte concentrations in P-PRP induce less tissue catabolism/inflammation than L-PRP treatment. In the present study, the difference of platelet concentrations between P-PRP and L-PRP was not significant, but rBMSC treated with L-PRP expressed less chondrogenesis-related genes, such as Col II, Aggrecan and Sox9, and their respective proteins compared with with P-PRP. Thus, the different effects of PRPs on the chondrogenesis of rBMSC may result from different leukocyte concentrations. In the present study, the nuclear translocation of NF-κB p65 was observed when rBMSC were cultured with L-PRP, and increased mRNA expression levels of iNOS, COX-2 and production levels of PGE2 and NO were determined, showing that the NF-κB pathway was highly activated and may impede the chondrogenesis and anabolism of rBMSC. It was reported that PRP releasate inhibited the inflammatory process in osteoarthritic chondrocytes through the reduction of IL-1β-induced NF-κB activation^43^; thus, the NF-κB pathway plays an important role in the cartilage repair. Pereira *et al*. reported that PRP lysate could contribute to the down-modulation of the NF-κB pathway and the COX-2 expression, thereby triggering the resolution of inflammatory responses^44^. However, neither platelet lysate nor PRP clot releasate contains viable leukocytes or platelets, while the excess of leukocytes may overwhelm the ability of growth factors on modulating pro-inflammatory cytokines^45^. Therefore, these studies may not reflect the effects of PRP on the NF-κB pathway. The findings of the present study demonstrated that L-PRP promoted the NF-κB pathway activation, which we reasonably ascribe it to the leukocytes in L-PRP. Leukocytes were significantly associated with an increased concentration of catabolic cytokines in PRP, including collagen-degrading MMPs, reactive oxygen species, and other catabolic proteases^46^. MMP-9 has been demonstrated to be a component of non-healing or poorly healing wounds^47^ and it was of high concentration in L-PRP, positively correlated with leukocytes^46^.

The harmful effect of leukocytes in PRP on chondrogensis *in vitro* may not occur *in vivo*. However, there has been no *in vivo* study that compares the cartilage repair in rabbit osteochondral defects between L-PRP and P-PRP. In our study, the rBMSC-containing P-PRP-derived scaffolds yielded better histological results than the rBMSC-containing L-PRP-derived scaffolds at 6 and 12 weeks after surgery. In addition, the interface of the joint surface in the PRP groups showed better integration compared with the control group, and that in the P-PRP group presented great integration. Valderrabano *et al*. reported that that chondral damage and poor cartilage integration promotes synovial fluid inflow, leading to cystic change seen in clinical studies^48^. Therefore it is important of the interface integration for the cartilage repair. PRP may enhance cartilage repair at the graft interface through recruitment of mesenchymal stem cells^49^. The introduction of rBMSC in PRP scaffolds may thus facilitate the recruitment efficiency.

However, the present study has certain limitations. Initially, we only preliminarily explored the mechanism of the different effects of L-PRP and P-PRP on rBMSC *in vitro* experiments, and the precise principle was not confirmed. Second, we evaluated the *in vivo* effects at 6 and 12 weeks, but did not observe the long-term effects. To solve the problems mentioned above, further studies that evaluate the effects of L-PRP and P-PRP on the NF-κB pathway *in vivo* are needed, and the observation period of *in vivo* studies should be prolonged. Third, the rabbit model of cartilage defects was not the same as the human cartilage lesion. The defects were created in the non-weight-bearing sites in our study, while most cartilage lesions occurred in the weight-bearing sites in clinics. Thus, we did not investigate the mechanical properties in this study.

In conclusion, the results suggest that both P-PRP and L-PRP can exert a beneficial effect on the cartilage repair, but P-PRP promotes better rBMSC in chondrogenic differentiation *in vitro* and *in vivo* compared with L-PRP. These findings also revealed that the better behavior of P-PRP in cartilage regeneration reflects the removal of the leukocytes reduces the concentrations of IL-1β and TNF-α and inhibits the activation of the NF-κB pathway, which affects the repair of cartilage defects. Thus, P-PRP treatment may present a better alternative for cartilage repair.

## Materials and Methods

The Independent Ethics Committee and the Animal Care and Use Committee of Shanghai Jiao Tong University Affiliated Sixth People’s Hospital approved the protocols used in the present study. All methods were performed in accordance with the relevant guidelines and regulations.

### Preparations of rBMSC, L-PRP, P-PRP and transplanted constructs

rBMSC were harvested from 6-week-old New Zealand white rabbits as per the Cao *et al*. procedure^50^. In brief, bone marrow was aspirated from each side of the iliac crests, anticoagulated with 1000 U/mL preservative-free heparin, and filtered with a 70-mm filter mesh. The bone marrow was subsequently suspended in the α-modification of minimum essential medium (α-MEM, Sigma-Aldrich, St Louis, MO, USA) containing 10% FBS (Gibco, Carlsbad, CA, USA), 100 U/mL penicillin G (Gibco) and 0.1 mg/mL streptomycin (Gibco) at 37 °C in a humidified atmosphere with 5% CO_2_. The medium was changed after 48 hours to remove non-adherent cells and thereafter once every three days. The multi-potency characterization and surface marker identification of rBMSC were performed according to a previous study^51^,^52^. Cells at the fifth passage were used in the present study.

Approximately 18 mL of autologous whole blood was collected from each New Zealand white rabbit through the central auricular artery and anticoagulated with 2 mL acid-citrate dextrose solution A (ACD-A) to make 20 mL of anticoagulated whole blood. Approximately 19 mL of anticoagulated whole blood was used in the preparation of PRPs, and 1 mL of whole blood was retained to quantify the platelet, leukocyte and cytokine concentrations in whole blood.

L-PRP was prepared using a buffy coat-based double-spin centrifugation method^53^. Briefly, 8 mL of autologous whole blood was transferred to a 15-mL centrifuge tube and centrifuged at 250 *g* for 10 minutes at room temperature to separate the blood into three phases: erythrocytes at the bottom, buffy coat in the middle (rich in platelets, leukocytes and fibrinogen), and platelet-containing plasma at the top. Platelet-containing plasma and buffy coat were subsequently transferred to a new tube and centrifuged again at 1000 *g* for 10 minutes, during which most of the platelets, leukocytes and fibrinogen precipitated. The supernatant plasma was discarded and the precipitated platelets were resuspended in the residual plasma to obtain a total of 1 mL of L-PRP.

P-PRP was prepared using a plasma-based method developed in the laboratory that concentrates platelets and eliminates leukocytes and erythrocytes. In brief, 11 mL of anticoagulated whole blood was centrifuged at 160 g for 10 minutes at room temperature to separate platelet-containing plasma from erythrocytes and buffy coat (rich in leukocytes). Care was taken to prevent the buffy coat and erythrocytes contamination. Platelet-containing plasma was subsequently transferred to a new tube and centrifuged at 250 *g* for 15 minutes. The supernatant plasma was discarded and the precipitated platelets were resuspended in the residual plasma to obtain a total of 1 mL of P-PRP.

The PRP scaffolds and transplanted constructs were prepared as per the Xie *et al*. method^12^. In brief, 0.5 mL of cell culture medium was added into 0.5 mL of the whole blood, L-PRP, or P-PRP to form blood clot or PRP scaffolds within minutes. To prepare the transplanted constructs, rBMSC of the third passage were detached, centrifuged and resuspended in cell culture medium at a concentration of 2.0 × 10^6^ cells/mL. Subsequently, 0.5 mL of the cell culture medium (containing rBMSC) was mixed with PRPs to prepare the PRP scaffolds, and the rBMSC-containing PRP scaffolds were used as transplanted constructs.

### Quantification of platelets, leukocytes, and released cytokines in whole blood and PRPs

The whole blood analyses were performed using an automatic hematology analyzer (XS-800i, Sysmex, Kobe, Japan) to determine the platelet and leukocyte concentrations of whole blood and PRPs.

The PRP scaffolds and blood clots were prepared as described above, followed by incubation at 37 °C in a humidified atmosphere with 5% CO_2_ for 7 days, and centrifugation at 2800 *g* for 15 minutes to collect 0.5 mL of supernatant^54^. The collected supernatant was assayed to determine the concentrations of IL-1β, TNF-α, PDGF-AB, and TGF-β1 using ELISA according to the manufacturer’s instructions (Xitang, Shanghai, China).

### Cell proliferation assay

rBMSC were seeded onto 96-well plates at a density of 4,000 cells/well for 20 hours in α-MEM without FBS to allow the cells to adhere, and subsequently the cells were transferred to a medium supplemented with 10% (volume/volume) of FBS, L-PRP, or P-PRP. The concentrations of PRP were determined based on previous studies^12^. The proliferation of rBMSC was assessed after 1, 3, 5, and 7 days using CCK-8 (Dojindo, Kumamoto, Japan). Briefly, 10 μL of CCK-8 solution was added to each well containing 100 μL of medium and incubated for 3 hours; blank medium plus an equal amount of CCK-8 was set as the background. The absorbance value was measured using a microplate reader (Bio-Rad, Hercules, CA, USA) at 450 nm.

### *In vitro* chondrogenesis induction assessment

The *in vitro* chondrogenesis induction assessment of rBMSC was conducted as per Xie *et al*. and Zhu *et al*. procedure^12^,^55^. In brief, approximately 500,000 rBMSC were seeded in 25-cm^2^ flasks and cultured in α-MEM supplemented with 10% (volume/volume) of FBS, L-PRP, or P-PRP, or cultured in a commercially-available chondrogenesis differentiation kit (CDK, Invitrogen, Rockford, IL, USA). The medium was changed once in every three days in all the cultures. Three weeks later, the cells were collected for quantitative real-time polymerase chain reaction (qRT-PCR) analysis and Western blotting analysis to detect the messenger RNA (mRNA) and protein expression, respectively, of Col I, Col II, Aggrecan and Sox9.

Total RNA was extracted from rBMSC using TRIzol reagent (Invitrogen, Rockford, IL, USA), and measured for quantity and purity using NanoDrop 2000 (Thermo Fisher Scientific, Rockford, IL, USA) according to the manufacturer’s instructions. Reverse transcription was performed using the High Capacity Reverse Transcription kit (Invitrogen). qRT-PCR was subsequently performed using a TP800 system (Takara, Japan) with SYBR Premix Ex Taq (Takara). β-Actin was used as a housekeeping gene for normalizing the results of the study. All the data were analyzed using the ΔΔCt method^56^. The sequences of the primers used are listed in Table 1.

rBMSC were lysed using mammalian protein extraction reagent (Pierce, Rockford, IL, USA) supplemented with complete protease inhibitor. The total protein concentration was quantified using BCA Protein Assay Kit (Pierce) according to manufacturer’s instructions. The total protein was denatured at 95 °C for 5 minutes. 100 μg of total protein was assessed using sodium dodecyl sulphate-polyacrylamide gel electrophoresis (SDS-PAGE), and the proteins were subsequently transferred to a polyvinylidene fluoride membrane (PVDF, Millipore, Billerica, MA, USA), which was blocked with a low-fat milk protein solution. The membranes were incubated with anti-Sox9 antibody (Abcam Cambridge, MA, USA), anti-Aggrecan antibody (Abcam), anti-Col I (Merck, Whitehouse Station, NJ, USA), anti-Col II (Merck), or anti-GAPDH antibody (Cell Signaling Technologies, Danvers, MA, USA), followed by peroxidase-conjugated secondary antibodies. The blots were then subjected to chemiluminescence detection using ECL western blotting substrate (Pierce).

### Evaluation of the effects of PRPs on NF-κB pathway *in vitro*

rBMSC were seeded onto cell culture plates at the appropriate density and cultured in α-MEM supplemented with 10% FBS until a confluent layer was achieved. rBMSC were subsequently serum-starved for 24 hours and treated in medium supplemented with 10% of FBS, L-PRP, or P-PRP for either 1 hour (immunofluorescence staining) or 48 hours (all other experiments).

rBMSC were seeded onto a chamber slide (ibidi, Martinsried, Germany) at a density of 30,000 cells/mL and treated as described above. Subsequently, rBMSC were fixed with 4% paraformaldehyde for ten minutes, treated with 3% Triton-X for 10 minutes, blocked with 10% FBS for 2 hours, incubated with primary antibody (anti-NF-κB p65 antibody, Abcam) at 4 °C overnight, and incubated with Alexa Fluor-conjugated secondary antibody (Abcam) for 2 hours. The cell nuclei were counterstained with DAPI (Life Technologies, Rockford, IL, USA), and subsequently washed subsequently with PBS. The nuclear translocation of NF-κB p65 was observed using a Leica DMI6000 B with Leica AF6000 software.

rBMSC were lysed to extract total RNA and the mRNA expression was analyzed for iNOS and COX-2 using qRT-PCR according to the methods described above. The primer sequences for the target genes are listed in Table 1.

Nuclear protein extracts were prepared using NE-PER Nuclear and Cytoplasmic Extraction Reagents Kit (Thermo Fisher Scientific, Rockford, IL, USA) according to the manufacturer’s instructions. Western blotting was subsequently performed as described above using an anti-NF-κB p65 antibody (Abcam). The amount of PGE2 released into the medium was quantified using an ELISA kit (Xitang). The effects of PRPs scaffolds on NO production were measured using a NO Assay Kit ((Thermo Fisher Scientific, Rockford, IL, USA).

### Animal surgery

Twenty-seven male mature New Zealand white rabbits (weighing 2.5–3.0 kg) were enrolled in the present study. After anesthetization achieved, a lateral para-patellar skin was incised and the knee joint was exposed when the joint capsule was sliced open and the patella was extracted laterally. A full-thickness cylindrical cartilage defect of 5 mm in diameter and 3 mm in depth was generated in the patellar groove using a stainless steel drill. The 48 defects in 24 rabbits were left unfilled (the control group), or randomly filled with transplanted constructs prepared with L-PRP (the L-PRP group) or P-PRP (the P-PRP group). The remaining 3 rabbits, which were used as a positive control for Micro-CT analysis and as a reference in histological scoring assessments, were also surgically treated but without any defects creation. All the rabbits were housed in separate cages and allowed to move freely immediately after surgery. Then 12 rabbits were euthanized after 6 weeks postoperatively and the remaining 15 rabbits were euthanized after 12 weeks postoperatively to harvest the distal part of the femur. The samples were fixed with 4% paraformaldehyde for 72 h and subsequently photographed.

### Gross morphology

The rabbits were sacrificed after 6 and 12 weeks postoperatively. Femoral condyle samples were dissected and fixed with 4% paraformaldehyde for 72 h, and then photographed and evaluated according to the ICRS macroscopic assessment scores for cartilage repair^57^.

### Micro-CT scanning

Micro-CT (Skyscan, 1076 scanner, Kontich, Belgium) scanning was performed to evaluate the subchondral bone regeneration in the defects of samples harvested after 12 weeks postoperatively. The samples were fixed for 72 h and immobilized with the femoral axis perpendicular to the scanning plane. The image data was reconstructed using NRecon software (Version 1.5.1.4, Skyscan) to visualize the 3D representation of the regenerated bone. Based on the CT data, a cylindrical region of interest (ROI) was analyzed which corresponds to the original defect location. For comparison between groups, the extent of bone regeneration within the defect was presented as a BV/TV ratio. The 3 rabbits without osteochondral defects were scanned as a positive control.

### Histological analysis

The samples were decalcified 10% EDTA for 1 month at 37 °C and then embedded in paraffin and cut into 5-μm sections that were then stained with hematoxylin and eosin (H&E) for general histological evaluation and with Toluidine blue for cartilaginous matrix distribution evaluation. The regenerated tissue was graded and evaluated by three trained observers according to the OARSI scale.

The expression of Col I and Col II was analyzed by immunohistochemical staining. The sections were dewaxed in xylene and hydrated through graded ethanol solutions, followed by blocking with 1% bovine serum albumin. Then the sections were incubated with the primary antibodies (mouse clone, 1:1000; Merck) at 4 °C overnight. The secondary anti-mouse IgG was added to the sections after washing three times with PBS and incubated for 1 h at 37 °C. The staining was developed in diaminobenzidine solution, with hematoxylin counterstaining.

### Statistical analysis

All experiments *in vitro* were repeated 3 times. The data were analyzed using the Statistical Package for Social Sciences version 22.0 (SPSS, IL, USA). The data are presented as the means ± standard deviation (SD). One-way analysis of variance (ANOVA) and Bonferroni’s post hoc test, or independent-samples Student’s t test was performed for statistical analysis. Statistical significance was indicated at *p* < 0.01 in *in vitro* tests or *p* < 0.05 in *in vivo* tests.

## Additional Information

**How to cite this article**: Xu, Z. *et al*. Comparative evaluation of leukocyte- and platelet-rich plasma and pure platelet-rich plasma for cartilage regeneration. *Sci. Rep.* **7**, 43301; doi: 10.1038/srep43301 (2017).

**Publisher's note:** Springer Nature remains neutral with regard to jurisdictional claims in published maps and institutional affiliations.

## Supplementary Material

Supplementary Information

## Figures and Tables

**Table 1:** The primers sequences used for qRT-PCR.

Genes	Forward primer sequence (5′-3′)	Reverse primer sequence (5′-3′)
Sox9	AAGGGCTACGACTGGACGCTGGTG	AGGGCCGCTTCTCGCTCTCG
Aggrecan	GTCTACAGAACAGCGCCATCATT	GCGAAGCAGTACACGTCATAGGT
Col II	TCGGCCTCCCTGGTATTGACG	GGAGGGCCCTGAGCACCATTGTT
Col I	AGCGTGGCCTACCTGGATGAAGC	ATGGGCGCGATGTCGGTGATGG
COX-2	CTTCACGCATCAGTTTTTCAAG	TCACCGTAAATATGATTTAAGTCCAC
iNOS	GCTGCCAAGCTGAAATTGA	GATAGCGCTTCTGGCTCTTG
β-Actin	GCATGGGCCAGAAGGACTCGTA	TCGCGGTTGGCCTTGGGGTTCA
