# Supplementary material for: Comparative evaluation of leukocyte- and platelet-rich plasma and pure platelet-rich plasma for cartilage regeneration
Source: Sci Rep. 2017 Mar 7;7:43301. doi: 10.1038/srep43301 (PMC5339695; doi:10.1038/srep43301)
Supplement: Supplementary Information [file srep43301-s1.pdf]

Comparative evaluation of leukocyte- and platelet-rich plasma and pure platelet-rich plasma for cartilage regeneration

Zhengliang Xu[1]*, Wenjing Yin[1]*, Yuelei Zhang[1], Xin Qi[1], Yixuan Chen[1], Xuetao Xie[1], Changqing Zhang[1]

1. Department of Orthopaedic Surgery, Shanghai Jiao Tong University Affiliated Sixth People's Hospital, Shanghai, China.

Zhengliang Xu and Wenjing Yin are co-first authors of this article.

Corresponding authors:

Changqing Zhang (e-mail: zhangcq@sjtu.edu.cn), Xuetao Xie (e-mail: xiexuetao1982@163.com).

Address: Department of Orthopaedic Surgery, Shanghai Jiao Tong University Affiliated Sixth People's Hospital, 600 Yishan Road, Shanghai 200233, People's Republic of China.

Telephone: +86-21-64369181

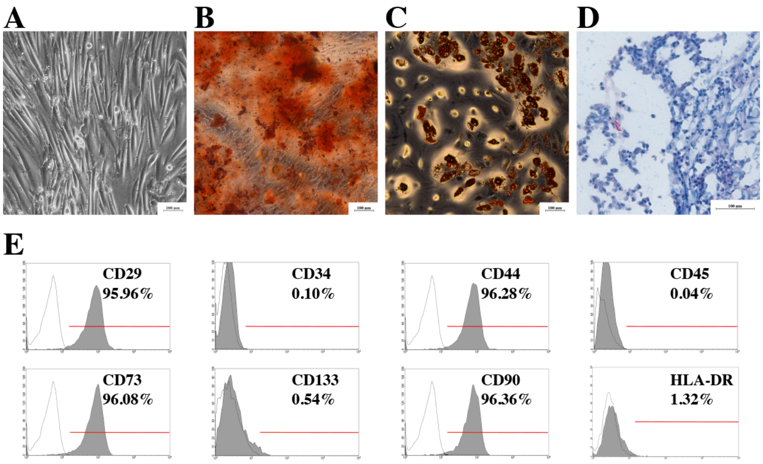

Figure S1. Identification of rBMSC. **(A)** microscopic appearance of rBMSC; **(B)** osteogenic differentiation of rBMSC was evaluated by alizarin red staining after osteogenic differentiation induction for 3 weeks; **(C)** adipogenic differentiation of rBMSC was evaluated by oil red O staining after adipogenic differentiation induction for 2 weeks; **(D)** chondrogenic differentiation of rBMSC was evaluated by alcian blue staining after chondrogenic differentiation induction for 4 weeks; **(E)** surface marker identification was performed by flow cytometry.

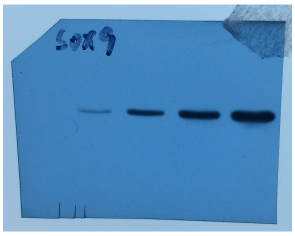

Figure 3B-Sox9

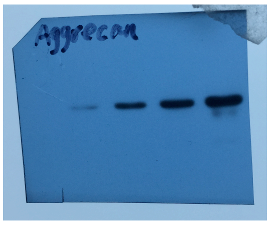

Figure 3B-Aggrecan

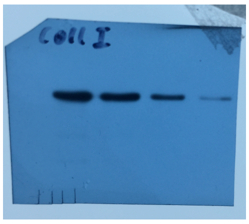

Figure 3B-Col I

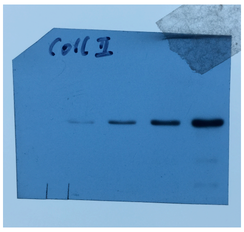

Figure 3B-Col II

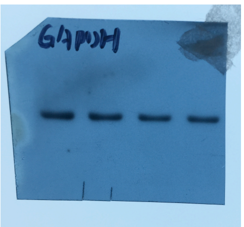

Figure 3B-GAPDH

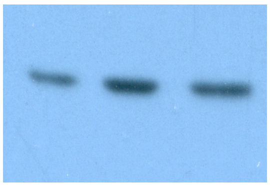

Figure 4B-NF-κB p65

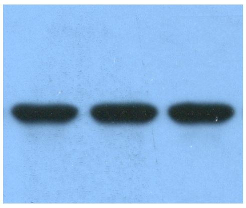

Figure 4B GAPDH
